# pH Tunes the DNA Repair Efficiency and Strand Preference of the AlkB Family Enzymes

**DOI:** 10.1021/acs.chemrestox.6c00202

**Published:** 2026-06-08

**Authors:** Samuel D. Howarth, Quentin J. Mylie, Evans Boateng-Boakye, Vincent Falkowski, Ambar Oliver Mella, Rafael Fermin, Zhiyuan Peng, Xin Bush, Yi-Tzai Chen, Jian Ma, Bongsup Cho, Deyu Li

**Affiliations:** Department of Biomedical and Pharmaceutical Sciences, College of Pharmacy, 4260 University of Rhode Island, Kingston, Rhode Island 02881, United States

## Abstract

AlkB-family Fe­(II)/2-oxoglutarate-dependent dioxygenases repair alkylated nucleic acid lesions through oxidative dealkylation and play important roles in genome maintenance. 1-Methyl-2′-deoxyadenosine (1mA) and 3-methyl-2′-deoxycytidine (3mC) are well-established substrates of AlkB, ALKBH2, and ALKBH3. Although these enzymes have been extensively studied, the influence of proton concentration (pH) on their catalytic behavior and strand preference remains poorly defined. Here, we systematically examined how pH modulates the activity of the prototypical bacterial AlkB and the human homologues ALKBH2 and ALKBH3 using defined DNA substrates in both single-stranded (ssDNA) and double-stranded (dsDNA) contexts containing 1mA and 3mC lesions. Across a broad pH range, all three enzymes mainly exhibit bell-shaped activity profiles with distinct optima. The prevailing view in the field is that AlkB preferentially repairs these lesions in ssDNA, ALKBH2 favors dsDNA, and ALKBH3 prefers ssDNA. However, our results demonstrate that pH influences the catalytic efficiency and strand utilization in a substrate- and enzyme-dependent manner. AlkB maintains a consistent ssDNA preference for 3mC but exhibits variable strand preference for 1mA at different pH values. ALKBH2 retains a strong dsDNA preference for 1mA across all conditions but shows a clear pH-dependent strand switch for 3mC, favoring ssDNA under acidic conditions and preferring dsDNA at neutral to alkaline pH conditions. In contrast, ALKBH3 consistently favors ssDNA for 3mC but exhibits pH-dependent strand preference for 1mA. Our results show that the reported strand preferences largely hold at pH 7.0–8.0 but are not complete, as strand utilization and pH optima vary by enzyme and substrate. The observations demonstrate that proton availability strongly influences AlkB-family catalysis and is an important factor in how these enzymes process damaged DNA. These findings may also aid the optimization of AlkB-based protein engineering and sequencing technologies.

## Graphical abstract

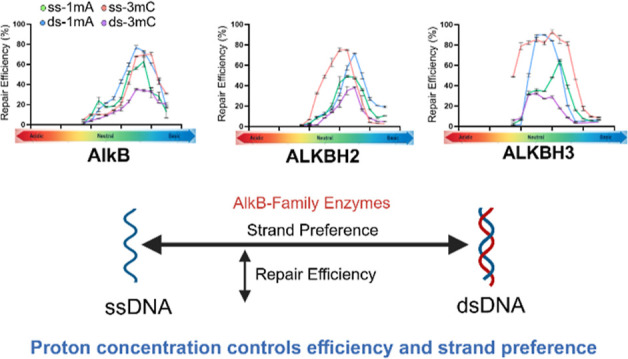

## Introduction

Cells of living organisms are constantly exposed to harmful agents, many of which can damage macromolecules such as DNA and RNA.^1,2^ These damaging agents arise from both endogenous processes and exogenous sources such as environmental toxins, tobacco smoke, and chemotherapeutic agents.^3,4^ Among the most prevalent forms of nucleic acid damage is alkylation, which encompasses many chemically diverse modifications that can be harmful in nature.^3,5,6^ Aberrant methylation can lead to the formation of adducts such as 1-methyl-2′-deoxyadenosine (1mA) and 3-methyl-2′-deoxycytidine (3mC) (Figure 1a), which disrupt Watson–Crick base pairing and are strongly cytotoxic and mutagenic.^3,7–11^

Because DNA carries the genetic information necessary for inheritance, preserving its integrity is essential for cellular and organismal survival.^3,12^ As a result, cells have evolved multiple repair pathways to remove alkylation damage and maintain nucleic acid integrity. One of these pathways is direct reversal repair (DRR),^7,8,13^ which restores modified bases without cleaving the nucleic acid backbone (Figure 1b).^3,6,7,13^ A major branch of this pathway is mediated by the Fe­(II)/2-oxoglutarate (2-OG)-dependent dioxygenases, whose members remove alkyl lesions through oxidative dealkylation (Figure 1b). The *Escherichia coli* (*E. coli*) AlkB protein is the prototypical and best-characterized member of this family and has served as the mechanistic model for understanding catalytic strategy, substrate recognition, and biological function across many homologues.^3,6,14,15^ Homologues of AlkB are widely distributed in nature,^3^ and mammalian cells encode nine known homologues: ALKBH1-8 and FTO.^16–20^ These enzymes share a conserved catalytic core, but they exhibit markedly distinct substrate specificities, strand preferences, and biological functions.^21–23^

**Figure 1:**
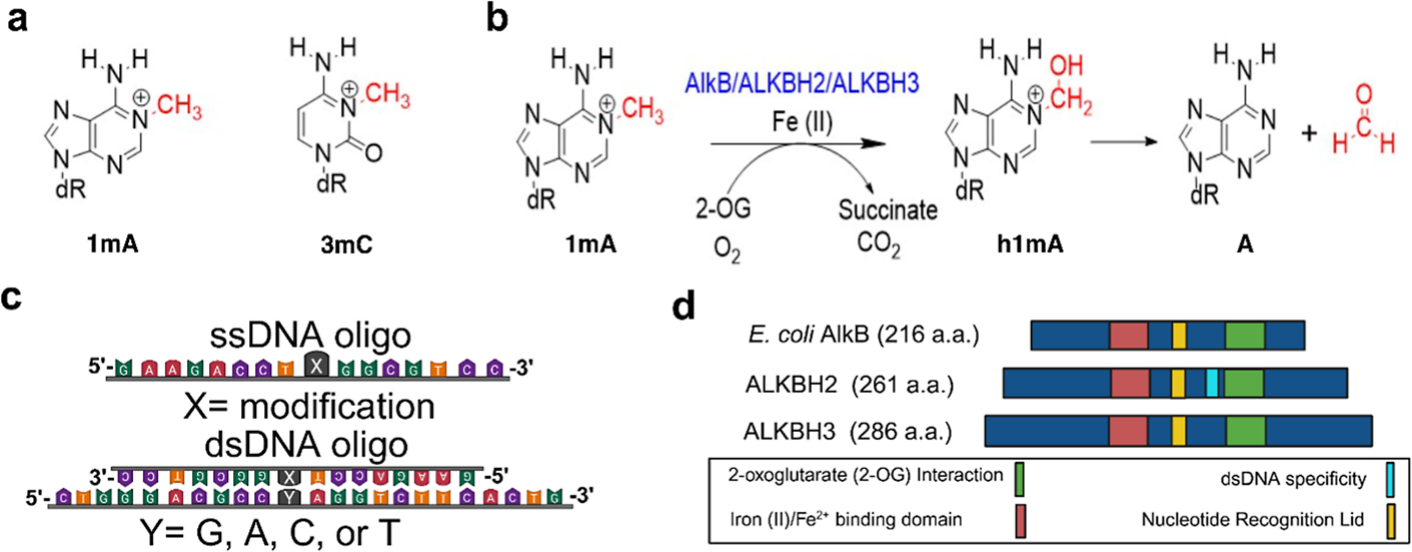
Substrates, DNA sequences, reaction chemistry, and domain organization of the AlkB-family enzymes. (a) Structures of the DNA modifications examined: 1mA and 3mC. (b) General mechanism of the AlkB-family oxidative demethylation represented with 1mA. (c) ssDNA and dsDNA substrate design. And (d) domain architecture of *E. coli* AlkB and human AlkB homologues (ALKBH2 and ALKBH3) used in this study showing the conserved active site domain.

Among the nine mammalian homologues, ALKBH2 and ALKBH3 are the best characterized DNA repair enzymes and are the two homologues most closely associated with reversal of alkylation damage in DNA like *E. coli* AlkB (Figure 1d).^16,17^ Both enzymes repair lesions such as 1mA, 3mC (which are also among the most efficiently processed substrates in the AlkB family)^15,24^ and others (Table S1).^3,25^ The high reactivity on 1mA and 3mC has been attributed in part to their cationic character under physiological conditions, which is thought to favor productive oxidative demethylation.^26,27^

Despite catalyzing closely related substrates, however, ALKBH2 and ALKBH3 are functionally distinct.^23^ ALKBH2 is predominantly localized in nucleus and is generally regarded as the principal mammalian AlkB-family enzyme for repairing lesions in double-stranded DNA (dsDNA). Whereas ALKBH3, similar to AlkB, has more been associated with single-stranded DNA (ssDNA) and RNA substrates, and is found in both the nucleus and cytoplasm (Figure 2).^16,17,22,28–31^ These differences suggest that regulatory factors beyond catalytic core conservation help define how individual AlkB-family enzymes process damaged nucleic acids.

**Figure 2:**
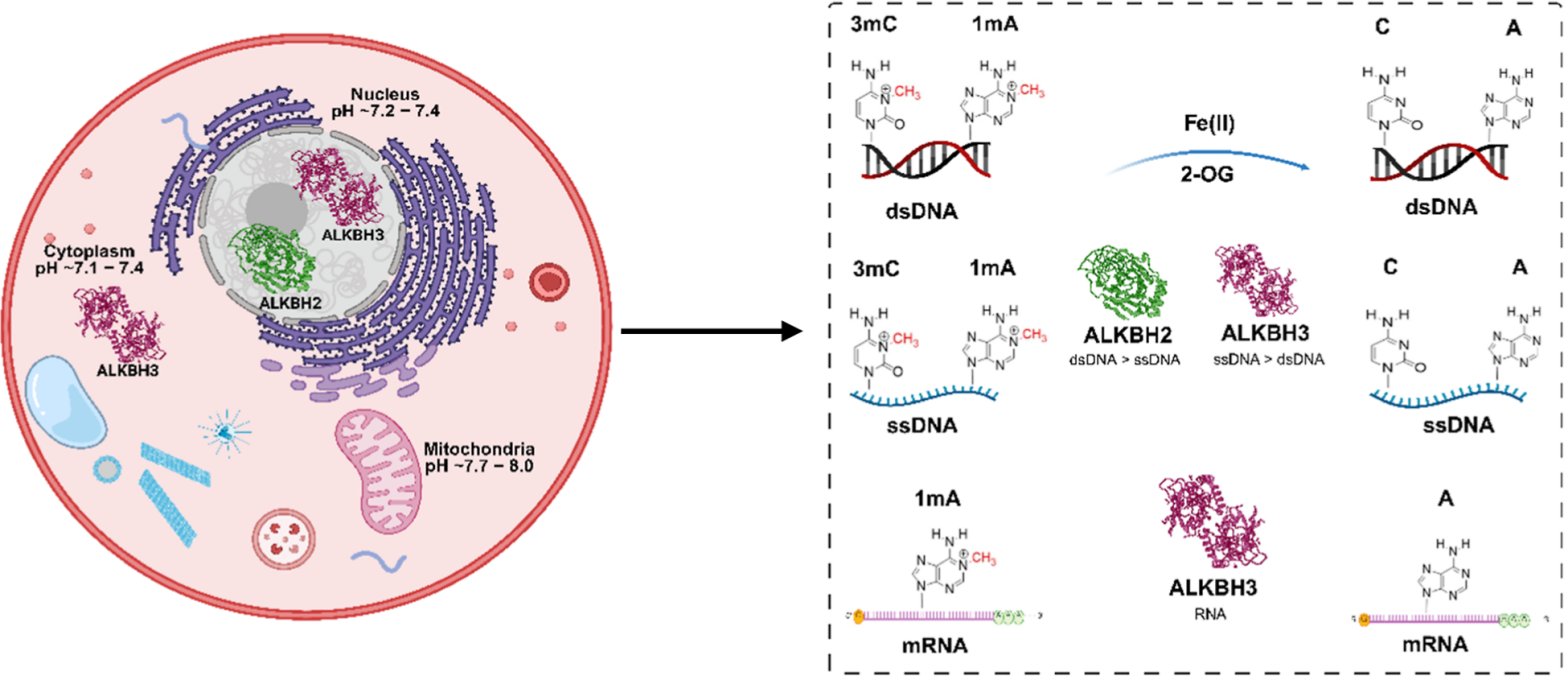
Substrate scope, strand preference, and subcellular context of ALKBH2 and ALKBH3. Subcellular localization and approximate compartmental pH ranges are indicated (left panel). Schematic of ALKBH2- and ALKBH3-mediated oxidative demethylation of alkylated nucleic acid lesions. ALKBH2 preferentially repairs lesions in dsDNA, whereas ALKBH3 favors ssDNA and can also act on RNA (right panel).

One potentially important but underexplored factor is proton concentration (pH). Intracellular pH is not uniform across cellular compartments, nor is it static under physiological or pathological conditions even though it is tightly regulated. Nuclear, cytoplasmic, and other subcellular environments maintain characteristic but dynamic proton environment that can shift in response to metabolism, oxidative stress, hypoxia, and disease.^32,33^ Such changes are chemically meaningful for AlkB-family enzymes because their catalytic cycle depends on precise Fe­(II) coordination, productive 2-OG binding, oxygen activation, correct positioning of a flipped nucleobase within the active site.^34–37^ Any of these steps could, in principle, be influenced by protonation state. In addition, the lesions themselves are chemically distinct and may respond differently to changes in proton availability, raising the possibility that pH affects both how efficiently these enzymes react and how they discriminate among substrates and strand contexts.

Strand preference is usually regarded as a relatively fixed property of the AlkB-family enzymes. In the published results, AlkB and ALKBH3 are commonly described as favoring ssDNA, whereas ALKBH2 is viewed as a dsDNA-preferring enzyme.^7,8,21,38^ However, most biochemical characterizations have been performed under a narrow range of standard assay conditions, typically near neutral pH (7.0–8.0), and systematic comparisons across broader pH windows remain unexplored. As a result, it remains unclear whether these strand preferences are truly invariant or instead represent conditional behaviors that depend on the chemical environment and substrate identity.

The influence of pH on DNA repair may also have broad biological relevance. Although intracellular pH in compartments associated with DNA repair is generally maintained within a relatively narrow range (∼7.1–8.0) (Figure 2), acid–base balance in cell and tissue is frequently perturbed in settings such as hypoxia, metabolic reprogramming, ischemia, inflammation, and cancer.^33,39,40^ Nonetheless, under tumor-associated acidosis, intracellular pH is typically buffered and rarely decreases below ∼6.8 before cellular viability is compromised.^40^ If AlkB-family enzymes are sensitive to such mild changes, then local proton availability could influence lesion persistence, repair efficiency, and possibly the balance between damage tolerance and mutagenesis. More broadly, because AlkB-family enzymes are increasingly used or engineered for applications in sequencing, nucleic acid modification mapping, and synthetic biocatalysis, understanding how pH shapes their catalytic behavior is important from a chemical and technological standpoint.

In this study, we systematically examined how pH influences the catalytic behavior of *E. coli* AlkB, ALKBH2 and ALKBH3 using 1mA- and 3mC-containing DNA substrates in both ssDNA and dsDNA contexts. By analyzing oxidative demethylation across a defined pH range (3.0–9.0) under standardized conditions, we asked whether pH simply modulates reaction rate or it also fundamentally alters strand preference and substrate processing. Although the full pH range examined here extends beyond the values expected for intracellular DNA repair compartments, such profiling provides a controlled biochemical approach to define pH tolerance, identify catalytic optima, and test whether strand preference is an invariant property or a condition-dependent behavior. Our results show that proton concentration influences the catalytic efficiency and also the strand preference in a lesion- and protein-dependent manner. In several cases, the reported strand preferences were maintained, but in others they were weakened, reversed, or rendered conditional by pH. These findings identify proton availability as an important factor influencing AlkB-family catalysis and strand behavior, and may aid the engineering of AlkB-based biochemical and sequencing applications.

### Experimental Procedure

#### Synthesis and Purification of Oligonucleotides

Single-stranded DNA (ssDNA) oligonucleotides (16-mer; 5′-GAAGACCTXGGCGTCC-3′, where *X* represents the modified nucleobase) were synthesized via automated solid-phase phosphoramidite chemistry using a MerMade-4 DNA synthesizer (LGC Biosearch Technologies/Bioautomation, Dexter, MI).^9,11,38^ Following synthesis, oligonucleotides were purified by high-performance liquid chromatography (HPLC) employing an anion-exchange DNAPac PA-100 semipreparative column (Phenomenex). The mobile phases consisted of deionized water (solvent A) and 1.5 M ammonium acetate in water (solvent B). DNA concentrations were quantified by UV absorbance at 260 nm. Molar extinction coefficients were calculated based on the corresponding unmodified sequence, as incorporation of a single modification within the 16-mer sequence produced negligible changes in the overall absorbance.

Double-stranded DNA (dsDNA) substrates were prepared by annealing the lesion-containing 16-mer strand with a 1.25-fold molar excess of complementary 23-mer oligonucleotides (5′-CTGGGACGCCYAGGTCTTCACTG-3′, where *Y* denotes T for 1mA, or G for 3mC; Integrated DNA Technologies). Annealing was performed under standard thermal cycling conditions to ensure complete duplex formation.

Oligonucleotide identity and integrity were characterized using an Orbitrap Exploris 240 LC–MS system (Thermo Fisher Scientific). Chromatographic separations were carried out on a Luna C18 column (4.6 × 100 mm, 5 μm particle size; Phenomenex) using a solvent system comprising 300 mM hexafluoro-2-propanol (HFIP) in water (solvent A) and 300 mM HFIP in methanol (solvent B). Observed monoisotopic masses and corresponding *m*/*z* values were compared with calculated values, and the results are summarized in Table S2.

### Expression and Purification of AlkB, ALKBH2, and ALKBH3

The coding sequence for *E. coli* AlkB was cloned into the pET28a expression vector (EMD Millipore), which introduces an N-terminal His_6_-tag to facilitate purification. The construct was transformed into *E. coli* Rosetta2­(DE3)­pLysS cells (EMD Millipore) for recombinant expression.^9,41^ A single colony was used to inoculate LB medium supplemented with kanamycin, and cultures were grown at 37 °C with shaking until reaching an optical density at 600 nm (OD_600_) of approximately 0.6–0.8. Protein expression was induced with 0.5 mM isopropyl β-D-1-thiogalactopyranoside (IPTG), and cultures were incubated for an additional 6 h at 30 °C to promote soluble protein expression.

Cells were harvested by centrifugation and resuspended in lysis buffer (50 mM *Tris*–HCl, 300 mM NaCl, 10 mM imidazole, and 1 mM 2-mercaptoethanol, pH 8.0). Following cell disruption by French Press, the lysate was clarified by centrifugation, and the supernatant was applied to a nickel-affinity HisTrap HP column (GE Healthcare Life Sciences) pre-equilibrated with lysis buffer. After extensive washing to remove nonspecifically bound proteins, AlkB was eluted using an imidazole gradient. The His-tag was removed by overnight thrombin digestion (0.005 U per μg of protein; Sigma-Aldrich) at 4 °C. The cleaved protein was further purified by cation-exchange chromatography using a HiTrap SP HP column (GE Healthcare Life Sciences). Final purified AlkB was concentrated using Amicon ultrafiltration devices (EMD Millipore) and stored in buffer containing 10 mM *Tris*, 100 mM NaCl, 1 mM 2-mercaptoethanol, and 10% glycerol (pH 8.0).

Human ALKBH2 and ALKBH3 were expressed and purified using a similar strategy. Expression constructs were transformed into *E. coli* BL21­(DE3)­pLysS cells (EMD Millipore).^38,41^ Cultures were grown at 37 °C to an OD_600_ of 0.4–0.8 and induced with 0.5 mM IPTG, followed by 6 h expression at 30 °C. Cells were lysed under comparable conditions, and proteins were purified via nickel-affinity chromatography, followed by cation-exchange chromatography for further refinement. Purified ALKBH2 and ALKBH3 were concentrated and stored in buffer containing 50 mM *N*-[*tris*(hydroxymethyl)­methyl]-3-aminopropanesulfonic acid (TAPS), 300 mM NaCl, 10% glycerol, and 1 mM 2-mercaptoethanol (pH 8.0). Protein identity and purity were determined using SDS PAGE- gel electrophoresis and a Bio-Rad protein standard (Figure S1).

### Preparation of a Universal Buffer System for pH-Dependent Enzymatic Assays

To evaluate enzyme activity across a broad pH range, a universal buffer system was employed to maintain consistent ionic composition while allowing controlled variation of proton concentration. The buffer consisted of a mixture of three buffering agents: 2-(*N*-morpholino)­ethanesulfonic acid (MES), 4-(2-hydroxyethyl)-1-piperazineethanesulfonic acid (HEPES), and sodium acetate. These components were selected because their combined buffering capacities span a wide pH interval. Each buffer component was incorporated at equimolar concentrations, yielding a final total buffering concentration of 60 mM in the reaction mixture.

The buffering performance of the universal system was verified by titrating an initially basified buffer solution with acid while monitoring pH changes at defined intervals. Incremental pH measurements confirmed that the combined buffer mixture maintained stable buffering capacity across the desired experimental range (Figure S2).

In vitro AlkB, ALKBH2, and ALKBH3 demethylation assays were conducted using this universal buffer system to systematically examine enzyme activity across varying pH conditions while maintaining consistent buffer composition. To ensure that the observed enzymatic behavior was not influenced by the specific buffering agents used, control reactions were also performed using individual buffer salts under comparable conditions. Desired pH values were obtained by adjusting buffer solutions with either hydrochloric acid or sodium hydroxide prior to use.

### In-Vitro Oxidative Demethylation Reactions

Oxidative demethylation assays with AlkB, ALKBH2, and ALKBH3 were carried out using conditions adapted from previously reported procedure.^24,38,42,43^ Reactions were performed with defined ssDNA or dsDNA oligonucleotide substrates containing the indicated modifications at a final substrate concentration of 5 μM. Unless otherwise noted, assays were conducted in 20 μL reaction volumes at 37 °C and contained 70 μM Fe­(NH_4_)_2_(SO_4_)_2_·6H_2_O, 0.93 mM α-ketoglutarate, 1.86 mM ascorbic acid, and 46.5 mM buffer adjusted to the desired pH.

For pH-dependence experiments, reactions were performed over a pH range of 3.0–9.0 under otherwise identical conditions. Individual reactions were allowed to proceed for 1 h. For each enzyme, the same enzyme concentration was used across all substrates tested within that experimental set, as summarized in Table S3. Reactions were quenched by addition of EDTA to a final concentration of 50 mM, followed by heating at 95 °C for 5 min to terminate enzyme activity. All assays were performed in quadruplicate. Following quenching, reaction mixtures were purified using a DNA Clean & Concentrator kit (Zymo Research) to remove proteins, metal ions, and small-molecule cofactors prior to downstream HPLC analysis.

### HPLC-Based Analyses of Enzymatic Reactions

To determine reaction end products and quantify the extent of substrate oxidation at the different pH conditions tested, postreaction oligonucleotides were first cleaned up, concentrated, and then analyzed by HPLC.

For reactions involving 1mA- and 3mC-containing oligonucleotides, oxidation by AlkB-family enzymes results in direct demethylation and restoration of the corresponding unmodified bases. These reactions were therefore analyzed at the intact oligonucleotide level by separating substrate and product species directly by anion-exchange HPLC. In these cases, the difference in charge between the methylated starting material and the demethylated product enabled chromatographic resolution. HPLC analyses were performed on a Thermo Ultimate 3000 system (Thermo Fisher Scientific, USA) equipped with a DNAPac PA-100 column (4 × 250 mm; Thermo Scientific). Elution was carried out using a gradient of 1.5 M ammonium acetate, beginning with a 5 min gradient from 50% to 65%, followed by 2 min at 70% ammonium acetate. Oligonucleotides were detected by UV absorbance at 260 nm. The extent of substrate conversion was determined by integrating substrate and product peak areas and expressing product formation as a percentage of the total detected analyte.

### LC–MS Analyses of Enzymatic Reaction Products

To confirm the identities of the products observed in the HPLC assays, representative reactions from the pH 7.0 experimental set were further analyzed by LC–MS (Figure S3). These experiments served as an independent validation that the chromatographic peaks assigned as reaction products corresponded to the expected demethylated species.

Mass spectrometric analyses were performed using an Orbitrap Exploris 240 mass spectrometer (Thermo Fisher Scientific) equipped with an electrospray ionization (ESI) source operated in negative-ion mode. The spray voltage was set to 2000 V, nitrogen was used as the drying gas at a flow rate of 40 L/min, and the ion transfer tube temperature was maintained at 320 °C.

Chromatographic separation was carried out on a Luna C18 column (4.6 × 100 mm, 5 μm particle size; Phenomenex) at a flow rate of 0.2 mL/min. The mobile phases consisted of 300 mM HFIP in water (solvent A) and 300 mM HFIP in methanol (solvent B). Oligonucleotides were eluted using a linear gradient from 37% to 80% solvent B over 4 min, followed by 80 to 100% B for 0.5 min. The column was then held at 100% B for 2 min, returned to 37% B over 0.5 min, and re-equilibrated at 37% B for 4 min prior to the next injection. Data acquisition and analysis were performed using Thermo Scientific Xcalibur Qual Browser (version 1.5).

## Results

Because the AlkB-family enzymes catalyze oxidative demethylation through Fe­(II)/2-oxoglutarate-dependent chemistry that requires precise substrate positioning and base flipping, we reasoned that proton concentration could influence both the catalytic rate, substrate strand engagement and discrimination. In this study, we investigated the pH dependence of oxidative demethylation by *E. coli* AlkB and its human homologues ALKBH2 and ALKBH3, using defined DNA oligonucleotides (oligos) in both single- and double-stranded contexts containing the strong substrates 1mA and 3mC. Those two homologues are the most extensively characterized DNA repair enzymes and the only reported homologues capable of complementing the function of AlkB in vivo to protect cells from alkylation damage.^16,17^ We employed the previously reported in vitro oxidative dealkylation procedure of this family enzymes.^9,38,44^ Recombinant full-length AlkB, ALKBH2, and ALKBH3 were expressed in *E. coli* with purity shown in Figure S1. Oligos containing the site-specific adducts were synthesized, purified; and their purity and identity were characterized by HPLC and LC–MS, respectively (Figures S4 and S5).^43^ We used HPLC-based methods to examine the extent of the oxidation of these substrates in both strand contexts. To ensure rigorous and comparable analysis across conditions, a universal buffer system (pH 3.0–9.0) was adopted, maintaining consistent ionic composition while varying proton concentration. Representative results for different pH conditions are shown in Figures 3 and S6. Generally, we observed that all three enzymes (AlkB, ALKBH2, and ALKBH3), exhibited relatively bell-shaped pH-activity profiles (Figures 4 and 5),^45^ with individually distinct and characteristic optima depending on enzyme, substrate, and the nucleic acid strand context as described below.

**Figure 3:**
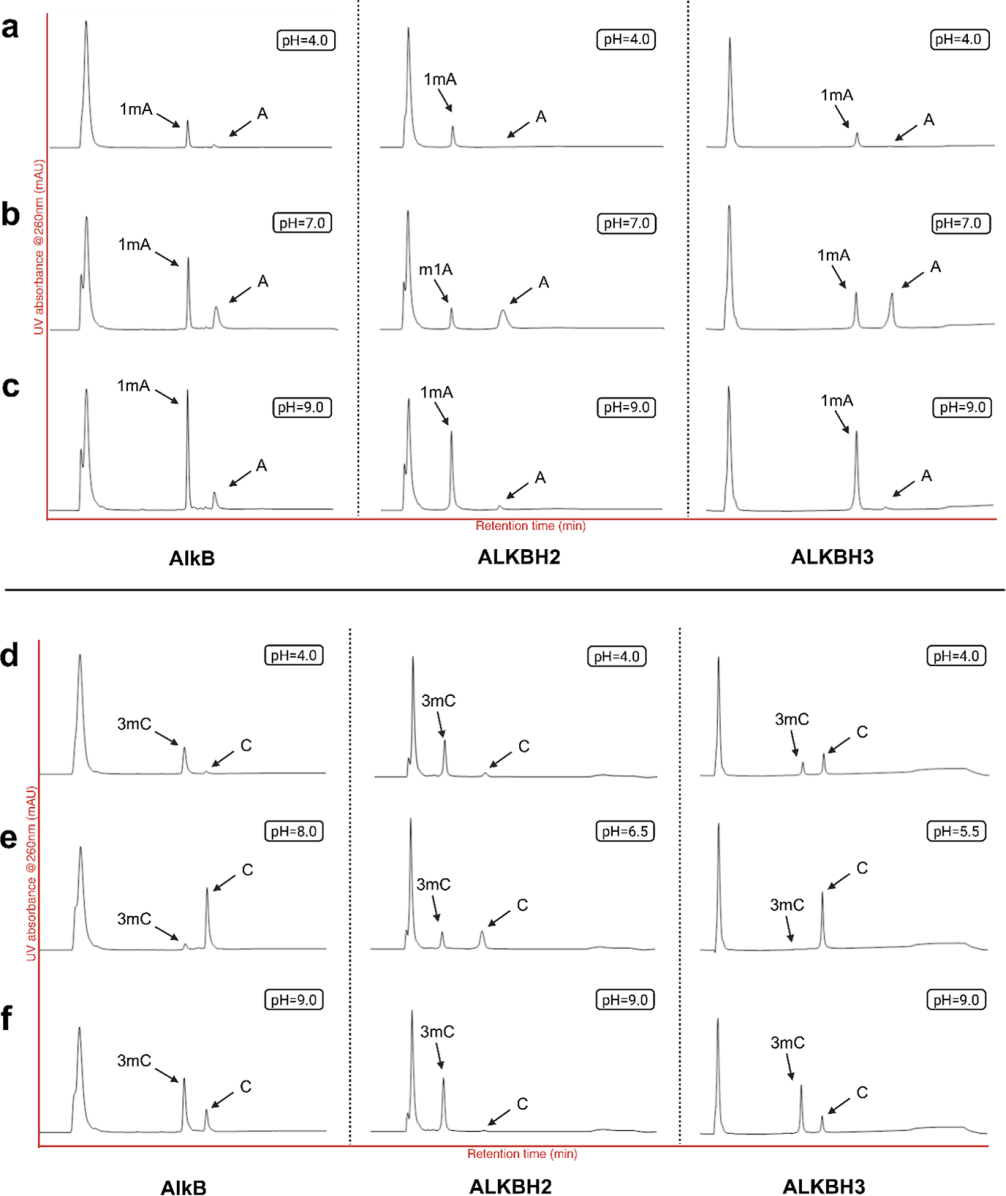
Representative HPLC traces of enzyme-mediated demethylation of ssDNA across pH conditions. (a–c) 1mA demethylation by AlkB, ALKBH2, and ALKBH3 at varying pH. (d–f) 3mC demethylation under different pH conditions. Peaks corresponding to substrate and product are indicated.

**Figure 4:**
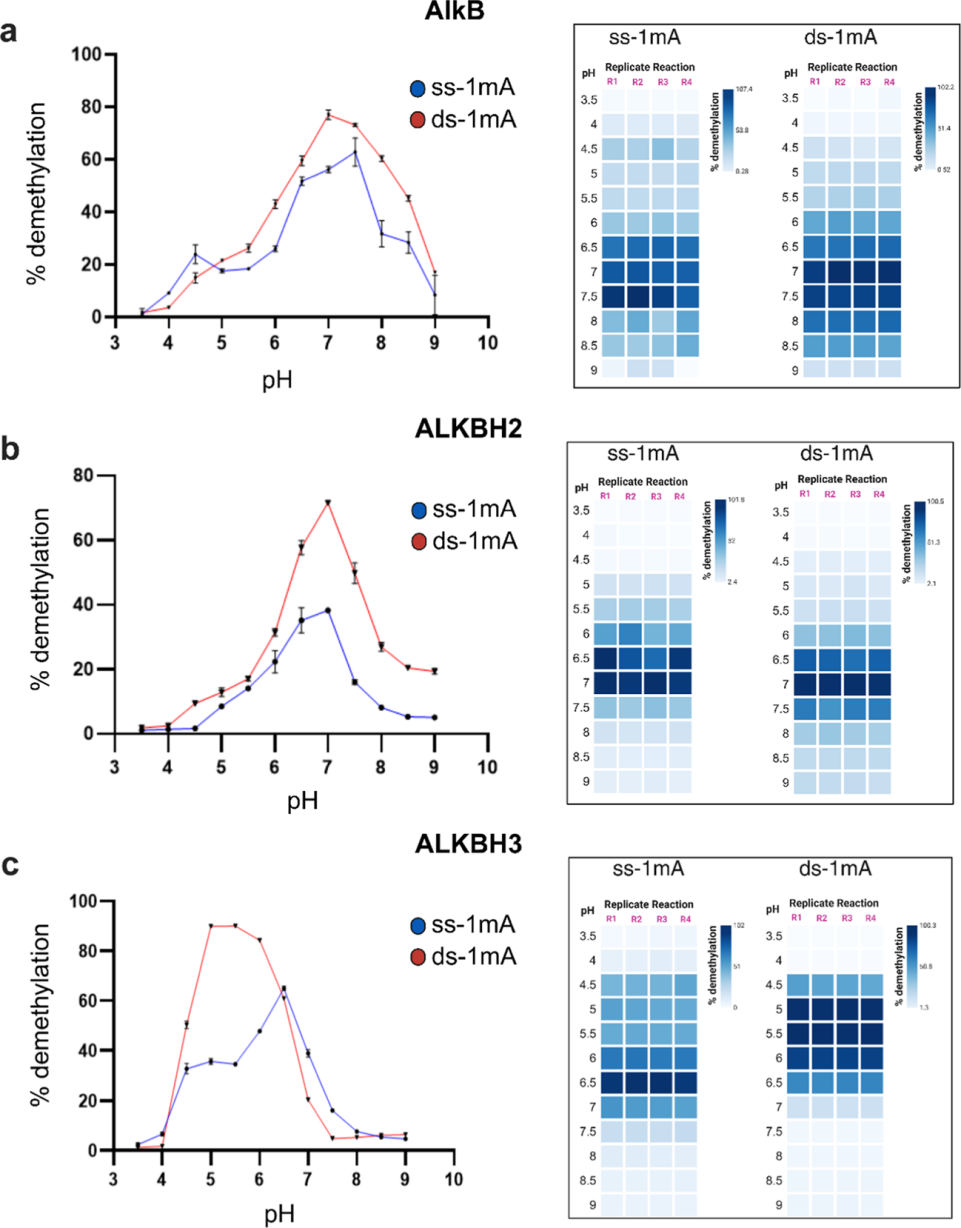
pH-dependent demethylation of 1mA by AlkB-family enzymes. (a–c) Percent demethylation of 1mA-containing ssDNA and dsDNA substrates by AlkB, ALKBH2, and ALKBH3 across the tested pH range. Line plots (left) show the mean percentage demethylation ± Standard Error of the Mean (SEM) from quadruplicate reactions. Heatmaps (right) show the corresponding replicate-level of demethylation for each pH condition, with each of the four columns representing one independent replicate reaction (*n* = 4) and each row representing a pH condition. The color scale indicates percent demethylation.

**Figure 5:**
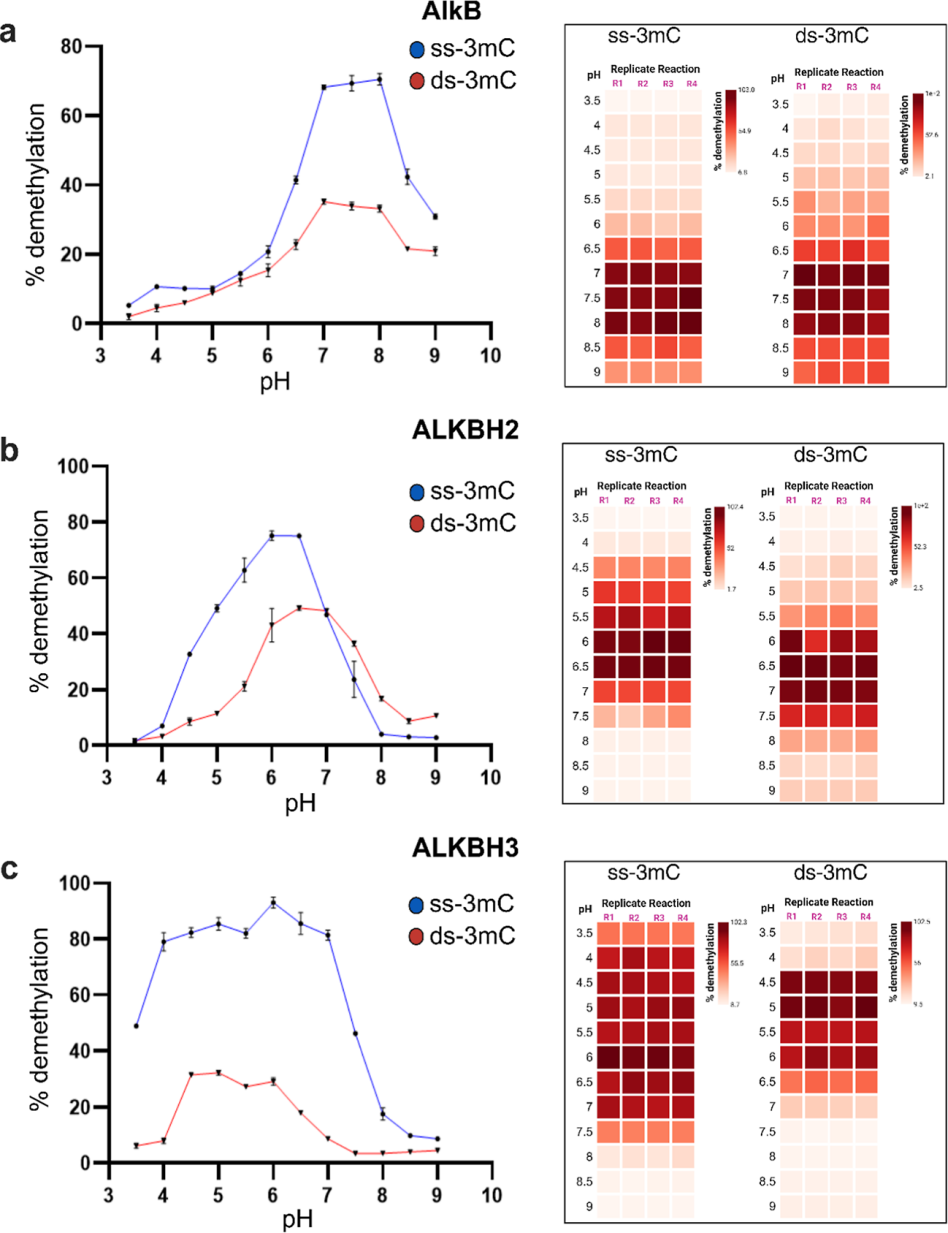
pH-dependent demethylation of 3mC by AlkB-family enzymes. (a–c) Percent demethylation of 3mC-containing ssDNA and dsDNA substrates by AlkB, ALKBH2, and ALKBH3 across the tested pH range. Line plots (left) show the mean percentage demethylation ± standard error of the mean (SEM) from quadruplicate reactions. Heatmaps (right) show the corresponding replicate-level of demethylation for each pH condition, with each of the four columns representing one independent replicate reaction (*n* = 4) and each row representing a pH condition. The color scale indicates percent demethylation.

### Part 1. AlkB Shows Broad pH Tolerance, but Its Strand Preference is Lesion-Dependent

AlkB retained strong activity over a relatively broad range near-neutral pH, with maximal demethylation of both 1mA- and 3mC-containing substrates centered approximately between pH 6.5 and 8.0 (Figures 4a and 5a; Table 2). For 1mA, both ssDNA and dsDNA were efficiently repaired, with the highest product formation occurring near pH 7.0–7.5 (Figure 4a). However, analysis of fold-difference for activity revealed that the strand preference for 1mA was not fixed. Under acidic conditions, AlkB retained modest ssDNA preference for 1mA, with ssDNA/dsDNA ratios of 2.58 ± 0.09 at pH 4.0 and 1.61 ± 0.16 at pH 4.5, but at higher pH this preference was lost and the enzyme showed comparable or modestly greater activity on dsDNA (Tables 3 and S4). Thus, under our assay conditions, AlkB did not maintain the canonical literature-reported ssDNA preference for 1mA across the full pH range tested.^3,15,38^

**Table 1:** Comparison of Literature-reported and Experimentally Observed Strand Preferences of AlkB-Family Enzymes for 1mA and 3mC^a^

		Experimental Observation	
enzyme	reported strand preference (1mA/3mC) pH 7.0–8.0	1mA	3mC
AlkB	ssDNA > dsDNA^3,15,25,38,44^	*dsDNA > ssDNA	ssDNA > dsDNA
ALKBH2	dsDNA > ssDNA^16,25,38^	dsDNA > ssDNA	**ssDNA > dsDNA (pH < 7.0)
ALKBH3	ssDNA > dsDNA^17,25,30,46^	**dsDNA > ssDNA (pH < 6.5)	ssDNA > dsDNA

a Reported strand preferences for AlkB, ALKBH2, and ALKBH3 on 1mA- and 3mC-containing substrates are summarized from prior studies performed under the commonly used pH range of 7.0–8.0. These published trends are compared with the strand preferences observed in the present study across the broader pH range examined. In several cases, the expected strand preference was maintained; however, pH-dependent deviations from the reported trends were also observed. The reversal in strand preference is marked with a single asterisk (*) to indicate that the difference between ssDNA and dsDNA repair was minimal and the two activities were nearly equivalent. Double asterisks (**) denote cases in which the observed deviation from the literature-reported strand preference was accompanied by a more pronounced difference in repair efficiency. For ALKBH2 on 3mC and ALKBH3 on 1mA, the pH thresholds below which these altered strand preferences became evident are indicated. These results highlight that strand preference within the AlkB family is not fixed but can shift depending on proton concentration and substrate context.

**Table 2:** Summary of Subcellular Localization, pH-Dependent Activity, and Strand Behavior of AlkB-Family Enzymes Examined in This Study^a^

enzyme	cellular localization	strand behavior	optimal pH window	substrate dependence
AlkB	bacterial cytosol	substrate-dependent	6.8–7.8	1mA: pH-dependent switch; 3mC: ssDNA > dsDNA
ALKBH2	nucleus	mixed/substrate-dependent	6.0–7.8	1mA: dsDNA > ssDNA; 3mC: pH-dependent switch
ALKBH3	nucleus, cytoplasm	mixed/substrate-dependent	4.5–6.5	1mA: pH-dependent switch; 3mC: ssDNA > dsDNA

a Cellular localization reflects the predominant intracellular distribution of each enzyme. The optimal pH window indicates the range over which maximal catalytic activity was observed as determined by pH-activity profiles. Strand behavior summarizes the relative preference for ssDNA or dsDNA, accounting for the pH-dependent and substrate-specific trends observed in this work. Unlike reported classifications, strand preference is not universally fixed across all conditions. AlkB exhibits substrate-dependent behavior, maintaining ssDNA preference for 3mC but showing varying strand preference for 1mA across pH. ALKBH2 consistently favors dsDNA for 1mA but displays a pH-dependent strand switch for 3mC, favoring ssDNA under acidic conditions and dsDNA at neutral to alkaline pH. ALKBH3 maintains a robust ssDNA preference for 3mC but exhibits variable, pH-dependent strand behaviors for 1mA.

**Table 3:** Comparative Fold-Difference Analysis of Strand Preference across pH^a^

	AlkB		ALKBH2		ALKBH3	
	1mA	3mC	1mA	3mC	1mA	3mC
pH	fold (ss/dsDNA)	fold (ss/dsDNA)	fold (ss/dsDNA)	fold (ss/dsDNA)	fold (ss/dsDNA)	fold (ss/dsDNA)
3.5	0.70 ± 0.32	2.61 ± 0.58	0.60 ± 0.04	0.79 ± 0.09	1.83 ± 0.32	8.05 ± 0.58
4.0	2.58 ± 0.09	2.38 ± 0.28	0.57 ± 0.11	2.16 ± 0.11	3.93 ± 0.21	10.05 ± 0.67
4.5	1.61 ± 0.16	1.70 ± 0.10	0.17 ± 0.01	3.82 ± 0.30	0.65 ± 0.02	2.62 ± 0.03
5.0	0.81 ± 0.02	1.14 ± 0.05	0.66 ± 0.03	4.30 ± 0.07	0.40 ± 0.01	2.65 ± 0.05
5.5	0.70 ± 0.02	1.16 ± 0.08	0.82 ± 0.02	2.97 ± 0.12	0.38 ± 0.00	3.02 ± 0.03
6.0	0.61 ± 0.02	1.35 ± 0.10	0.71 ± 0.06	1.75 ± 0.13	0.57 ± 0.00	3.20 ± 0.08
6.5	0.87 ± 0.02	1.81 ± 0.08	0.61 ± 0.04	1.53 ± 0.02	1.07 ± 0.01	4.79 ± 0.12
7.0	0.73 ± 0.01	1.94 ± 0.03	0.53 ± 0.01	0.97 ± 0.00	1.91 ± 0.04	9.47 ± 0.27
7.5	0.86 ± 0.04	2.05 ± 0.05	0.32 ± 0.01	0.65 ± 0.09	3.35 ± 0.06	13.83 ± 0.69
8.0	0.53 ± 0.04	2.13 ± 0.04	0.30 ± 0.01	0.24 ± 0.01	1.45 ± 0.05	5.13 ± 0.31
8.5	0.63 ± 0.05	1.97 ± 0.05	0.26 ± 0.00	0.35 ± 0.02	0.88 ± 0.03	2.49 ± 0.08
9.0	0.75 ± 0.18	1.48 ± 0.05	0.26 ± 0.01	0.26 ± 0.00	0.73 ± 0.02	1.96 ± 0.05

a Fold-differences were calculated as repair efficiency on ssDNA divided by that on dsDNA (ssDNA/dsDNA) for AlkB, ALKBH2, and ALKBH3 on 1mA- and 3mC-containing substrates, with propagated mean ± standard error of the mean (SEM) (*n* = 4 independent reactions). Values greater than 1 indicate ssDNA preference, whereas values less than 1 indicate dsDNA preference.

In contrast, AlkB behaved much more consistently on 3mC. Activity peaked within the near-neutral to mild alkaline range (Figure 5a), but fold-difference analysis showed that AlkB consistently preferred ssDNA over dsDNA for 3mC across the entire pH range examined, with ssDNA/dsDNA ratios remaining above 1 at all pH values (Tables 3 and S5). This ssDNA preference was particularly evident between pH 6.5 and 8.5, and extremely low pH values of 3.5 and 4.0 where the fold-difference ranged from approximately 1.8- to 2.6-fold. The AlkB data show that it retains a constant ssDNA preference for 3mC, but not for 1mA.

### Part 2. ALKBH2 Retains Established dsDNA Preference for 1mA but Shows a pH-Dependent Preference Switch on 3mC

We next examined ALKBH2, the major mammalian AlkB-family enzyme associated with repair of lesions in duplex genomic DNA. For 1mA, ALKBH2 displayed a clear and internally consistent dsDNA preference across the entire pH range evaluated (Figure 4b; Tables 3 and S6). Activity rose sharply from acidic conditions to a maximum near pH 7.0, after which it declined at more alkaline pH. At every pH value examined, the ssDNA/dsDNA ratio remained below 1, indicating that ALKBH2 maintained its reported strand preference for duplex DNA throughout. The strongest dsDNA preference was observed between approximately pH 7.0 and 9.0, where the fold-difference ranged from ∼0.53 to 0.26 (Table 3).

In contrast, ALKBH2 behaved very differently on 3mC. Although the overall activity profile remained bell-shaped, the strand preference was strongly pH-dependent (Figure 5b; Tables 3 and S7). Under acidic to near neutral conditions, ALKBH2 favored ssDNA, with ssDNA/dsDNA fold-differences of 2.16 ± 0.11 at pH 4.0, 3.82 ± 0.30 at pH 4.5, and 4.30 ± 0.07 at pH 5.0. This ssDNA bias gradually diminished as pH increased, approaching parity at pH 7.0 (0.97 ± 0.00), and then reversed at higher pH to favor dsDNA, with fold-differences falling below 1.0 at pH 7.5–9.0 (Tables 3 and S7). Thus, unlike 1mA, 3mC repair revealed a clear pH-driven strand switch in ALKBH2, with acidic conditions favoring ssDNA processing and neutral-to-alkaline conditions favoring dsDNA repair. This behavior is especially notable because ALKBH2 is generally classified as a dsDNA-preferring enzyme.

### Part 3. ALKBH3 Shows Variable Strand Preference on 1mA but Consistent ssDNA Preference on 3mC

Lastly, we analyzed ALKBH3, which is generally considered a ssDNA- and RNA-preferring mammalian AlkB homologue. For 1mA, ALKBH3 displayed a broad activity window extending from acidic to near-neutral pH, with maximal activity observed between approximately pH 5.0 and 6.5 (Figure 4c). However, the strand preference for 1mA was not uniform across this range. Fold-difference analysis revealed that ALKBH3 alternated between ssDNA-favored and dsDNA-favored behavior depending on pH (Tables 3 and S8). Under acidic pH, ALKBH3 initially favored ssDNA, with fold-differences of 1.83 ± 0.32 at pH 3.5 and 3.93 ± 0.21 at pH 4.0. This pattern then reversed between pH 4.5 and 6.0, where dsDNA was favored, with fold-differences ranging from 0.65 ± 0.02 to 0.38 ± 0.00. Near pH 6.5, activity became nearly equivalent between strand contexts (1.07 ± 0.01), after which ssDNA again became favored between pH 7.0 and 8.0, before a modest dsDNA preference re-emerged at the high pH values tested (Table S8). Thus, ALKBH3 on 1mA does not follow a consistent strand preference but instead exhibits a strongly pH-dependent and nonmonotonic strand response.

By contrast, ALKBH3 showed a much more coherent pattern on 3mC. Activity was high over a broad range extending from acidic to near-neutral pH, and fold-difference analysis demonstrated that ALKBH3 consistently preferred ssDNA over dsDNA for 3mC at every pH point evaluated (Figure 5c; Tables 3 and S9). The magnitude of this ssDNA preference was substantial, reaching 10.05 ± 0.67 at pH 4.0, 9.47 ± 0.27 at pH 7.0, and 13.83 ± 0.69 at pH 7.5, before declining at more alkaline pH while still remaining above 1.0 (Tables 3 and S9). The data demonstrate 3mC as a robust ssDNA-preferred substrate for ALKBH3 across changing proton conditions, in contrast to the highly pH-sensitive strand behavior observed for 1mA.

### Part 4. Comparison across Homologues Reveals that pH Modulates Strand Preference in a Lesion-Specific Manner

Altogether, these results show that pH could modulate AlkB-family reaction rate and also reshape strand preference and substrate behavior in a lesion-specific and homologue-specific manner. This is most clearly illustrated by comparing literature-reported strand preferences with the experimental observations in this study, as three distinct patterns emerged (Table 1).

First, some strand preferences remain consistent across pH, such as ALKBH2 on 1mA and ALKBH3 on 3mC, indicating that certain enzyme–substrate combinations are robust to variations in proton concentration. Second, some combinations show conditional or switched strand behavior, most notably ALKBH2 on 3mC, where the enzyme switched s from ssDNA preference at acidic pH to dsDNA preference at neutral and alkaline pH. Third, some combinations show oscillating behavior, exemplified by ALKBH3 on 1mA, where the preferred strand changes more than once across the pH range examined.

The summary comparison at the optimal or near-optimal conditions for each enzyme (Figure 6) and the broad pH heatmaps (Figures 4 and 5) together indicate that the influence of proton concentration is not conserved even between two established strong substrates, 1mA and 3mC. Instead, pH appears to function as a contextual factor that can either preserve, weaken, or reprogram strand preference depending on the specific enzyme–lesion pair.

**Figure 6:**
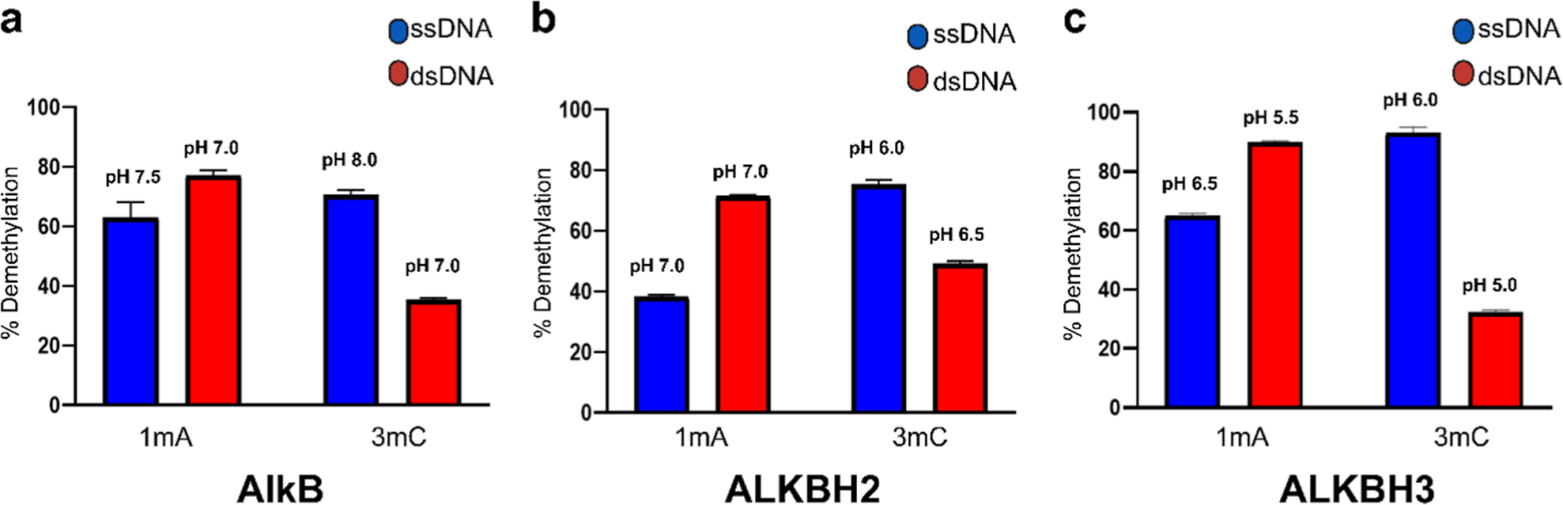
Comparison of strand preference at optimal pH for AlkB-family enzymes. (a–c) Percent demethylation/oxidation of 1mA and 3mC in ssDNA and dsDNA by AlkB, ALKBH2, and ALKBH3 at their respective optimal pH conditions. Bars highlight substrate-dependent strand preferences for each enzyme.

## Discussion

This study demonstrates that proton concentration is an important determinant of repair efficiency, pH optimum, and strand preference for AlkB-family enzymes. Although AlkB, ALKBH2, and ALKBH3 all catalyze Fe­(II)/2-oxoglutarate-dependent oxidative demethylation through a conserved chemical mechanism, our data show that the effect of pH on catalysis is not uniform across homologues or substrates. Instead, proton concentration correlates with lesion identity and strand context to shape how each enzyme engages and processes damaged DNA.

A common feature across all three enzymes was the presence of bell-shaped pH-activity profiles, indicating that each enzyme operates optimally within a defined protonation environment. Such behavior is expected for Fe­(II)/2-oxoglutarate-dependent dioxygenases, whose activity depends on proper coordination of the Fe­(II) center, productive binding of 2-oxoglutarate, oxygen activation, and precise positioning of the damaged base for oxidation. Perturbation of protonation states within the active site, on the substrate, or in surrounding structural elements would therefore be expected to influence one or more of these steps in catalysis. Importantly, our results show that pH affects more than overall catalytic efficiency. In several cases, proton concentration also altered strand preference, indicating that pH can influence substrate engagement and strand discrimination in addition to turnover itself. The comparatively large fold-difference values observed at certain pH points, particularly around pH 4.0, primarily reflect transient differences in repair efficiency between ssDNA and dsDNA substrates. The underlying trends of pH-dependent activity generally remain smooth within each strand context and do not show abrupt discontinuities.

One of the clearest conclusions from this work is that strand preference is not a fixed intrinsic property of these enzymes. This is especially evident for ALKBH2 on 3mC, where the enzyme switched from ssDNA preference at acidic pH to dsDNA preference at neutral and alkaline pH. ALKBH2 is widely described as a dsDNA-preferring repair enzyme, and the notion remains correct for 1mA, for which we observed dsDNA preference at every pH evaluated. However, our data show that this behavior does not generalize across all substrates. Instead, the strand preference of ALKBH2 depends on both lesion structure and proton concentration. This is an important mechanistic point because it suggests that duplex interrogation, base flipping, and productive lesion positioning in ALKBH2 are likely not governed by a single rigid structural preference but can be modulated by the chemical state of the substrate and reaction environment.

A related but distinct pattern was observed for ALKBH3 on 1mA, where strand preference was not simply switched once, but was instead varying across pH, alternating between ssDNA- and dsDNA-favored behavior. This was not the case for 3mC, for which ALKBH3 consistently preferred ssDNA across the full pH range examined. Thus, even with the same enzyme, pH can affect strand behavior very differently depending on the lesion being processed. These findings argue against a simplified model in which ALKBH3 is always intrinsically “ssDNA-preferring”. Rather, ALKBH3 is robustly ssDNA-preferring for 3mC, but not uniformly so for 1mA under all pH conditions.

The behavior of AlkB reinforces this extended conclusion. AlkB maintained a consistent ssDNA preference for 3mC, but not for 1mA, where the previously reported strand bias was only evident under acidic conditions and was diminished or reversed at high pH points. This means that even the bacterial prototype does not respond to pH in a unchanged way. Together, the results across all three enzymes show that 1mA and 3mC, despite both being strong substrates, are not functionally equivalent with respect to proton-dependent strand preference.

Why might these substrate-dependent differences occur? One likely explanation is that pH influences the balance between lesion charge-state, local electrostatics, and productive base positioning in a substrate-specific manner. Both 1mA and 3mC are positively charged under neutral condition, but they differ in ring structure, hydrogen-bonding potential, and their effects on nucleic acid stability in ssDNA versus dsDNA contexts.^26^ These differences could alter base-flipping energetics, lesion accessibility, and active-site accommodation in ways that become more or less favorable depending on proton concentration. In that sense, pH would not simply “tune the enzymatic activity up or down”, but could alter the relative energetic favorability of different enzyme–substrate–strand states.

Consistent with this idea, both 1mA and 3mC possess reported p*K*_a_ values of approximately 8.3 and 8.7, respectively.^47^ As pH transitions into the alkaline range, progressive deprotonation of these lesions may alter their charge distribution, hydrogen-bonding behavior, and interactions within the active site. Because the cationic character of 1mA and 3mC has been proposed to contribute to their efficient recognition and oxidation by AlkB-family enzymes,^26^ partial loss of protonation at basic pH condition may reduce favorable electrostatic interactions required for productive substrate recognition and catalysis. Thus, the decline in repair efficiency observed from pH 7 to 9 may reflect combined effects of enzyme protonation, cofactor binding as well as altered substrate ionization.

The unusual pH-dependent behavior observed for 3mC may also relate to the intrinsic tautomeric properties of the lesion itself. All canonical nucleobases, as well as their chemically modified counterparts, can undergo tautomerization to generate both major and minor tautomeric forms.^48^ The propensity for these conversions is governed by fundamental acid–base catalysis, in which proton transfer reactions facilitate interconversion between tautomeric states. In aqueous environments, water mediates rapid proton exchange, allowing nucleic acid tautomers to exist in dynamic equilibrium. Among the factors influencing this equilibrium, pH plays an important role by modulating protonation states and the relative stability of individual tautomers. Under physiological conditions, the canonical nucleobases predominantly exist in a single major tautomeric form. In contrast, chemically modified nucleic acids may display greater tautomeric diversity, particularly when their p*K*_a_ values are shifted toward physiological pH.^48^ Recent studies on m3C-containing RNA have shown that this modification can access alternative tautomeric states that influence its hydrogen-bonding properties and base-pairing behavior.^49^ Similar effects may occur in DNA and could alter duplex stability, lesion exposure, or base-flipping energetics in a pH-dependent manner. This possibility may be particularly relevant for the pronounced strand switching observed for ALKBH2 on 3mC and the strong ssDNA preference retained by ALKBH3 across a broad pH range.

Another consideration relevant to interpretation of 1mA reactivity at alkaline pH is the potential for Dimroth rearrangement of 1mA to *N* ^6^-methyl-2′-deoxyadenosine (6mA) under certain reaction conditions. It has been reported that thiol-mediated conversion of 1mA to 6mA can occur over extended incubation periods, particularly under mildly alkaline conditions.^50^ Our group has also previously reported that 6mA can be formed as a byproduct during the chemical synthesis of 1mA oligonucleotides under high temperatures and long deprotection time under alkaline conditions.^43^ Although trace carryover of reducing agents or thiols from protein purification cannot be completely excluded in the present study, several observations suggest that substantial Dimroth rearrangement was unlikely to influence the repair trends reported here for 1mA demethylation. First, shorter deprotection time and mild conditions were utilized in the 1mA oligonucleotide synthesis, after which we characterized its identity using HPLC and LC–MS techniques against 1mA and 6mA oligonucleotide standards. Also, the enzymatic reactions were performed at 37 °C for a maximum of 60 min, shorter than the multihour incubations reported to support efficient thiol-mediated conversion of 1mA to 6mA. Lastly, our HPLC analysis of reaction products showed retention–time profiles consistent with the expected 1mA-containing substrates and repaired adenine products, without evidence of additional peaks expected from substantial formation of 6mA-containing oligonucleotides (see Figures 3a–c, S4a and S6a–c). Because 1mA is positively charged whereas 6mA is neutral under these conditions, and because the tested AlkB-family enzymes (AlkB, ALKBH2, and ALKBH3) repair 6mA less efficiently than 1mA, substantial rearrangement to 6mA would be expected to reduce overall repair efficiency. In addition, significant formation of 6mA-containing oligonucleotides would likely produce distinct chromatographic behavior in our anion-exchange chromatography analyses relative to the standard 1mA oligonucleotide. Nevertheless, the possibility of partial Dimroth rearrangement under alkaline, high temperature or reducing conditions should be considered in studies examining 1mA chemistry in AlkB-family systems, particularly in long–duration reactions or engineered biochemical applications.

Furthermore, the local DNA sequence surrounding the lesion site may influence the observed repair efficiencies. It has been shown that AlkB-family enzymes can exhibit sequence-dependent differences in substrate recognition and catalytic efficiency,^38,51^ likely due to effects on local duplex stability, base stacking, lesion accessibility, and base-flipping dynamics. Because all substrates in the present study were examined within a single defined sequence context, the current study focused on the effects of pH, lesion identity, and strand context while minimizing sequence-dependent variability. Despite this, it remains possible that the magnitude of the pH-dependent effects observed here could vary in alternative sequence environments. Future studies examining broader sequence contexts may therefore provide additional insight into how local nucleic acid structure and proton availability collectively influence AlkB-family catalysis.

While the physiological implications of these observations remain to be fully established, our results demonstrate that proton concentration can meaningfully influence AlkB-family catalytic behavior under controlled biochemical conditions. Although intracellular pH in compartments associated with DNA repair is generally maintained within a relatively narrow range (∼7.1–8.0) (Figure 2), modest fluctuations can occur during metabolic stress, hypoxia, altered respiration, and disease states such as cancer.^33,39,40^ Even under tumor-associated acidosis, intracellular pH is typically buffered and rarely decreases below ∼6.8 before cell viability is compromised.^40^ Our in vitro findings do not directly establish how proton availability, especially outside physiological pH ranges, affects repair efficiency and strand utilization in vivo. Although the pH range examined here extends beyond that expected for intracellular DNA repair compartments, it provides a controlled way to define pH tolerance, identify catalytic optima, and determine whether strand preference is fixed or condition-dependent. In particular, the strongly acidic conditions examined here are best viewed as biochemical expansions of enzyme activity and substrate-dependent pH sensitivity rather than direct models of DNA repair in cells. The most biologically relevant implications of our findings likely lie within the near-neutral to mildly acidic range, where modest intracellular pH shifts may influence enzyme behavior, whereas the broader pH profile mainly informs biochemical mechanism and tool optimization.

Our data suggest that strand preference in AlkB-family enzymes may not be universally fixed, as perceived, but can depend on the combined effects of substrate identity and protonation environment. For example, we found that ALKBH2 may be less strictly duplex-preferred for certain lesions under acidic conditions than generally assumed, whereas ALKBH3 may maintain strong ssDNA preference for some lesions but not others. Beyond possible cellular implications, these findings are very relevant from a biochemical and technological perspective. AlkB-family enzymes are increasingly engineered for applications including nucleic acid modification mapping, sequencing technologies, substrate-selective oxidation, and synthetic biocatalysis. Most engineering strategies have focused on active-site mutations or structural redesign to alter substrate scope and reactivity. Our results suggest that proton concentration represents an additional component capable of influencing catalytic efficiency and strand preference of these enzymes. Consideration of pH-dependent effects may therefore improve interpretation of in vitro assays and aid optimization of engineered AlkB-systems designed for non-native substrates or reaction environments.

Several limitations should be mentioned for this study. These experiments were performed using defined oligonucleotide substrates under controlled in vitro conditions and therefore do not fully capture the complexity of chromatin organization, interacting protein partners, or intracellular microenvironments. In addition, although the present data define clear pH-dependent substrate and strand behavior of these enzymes, they do not yet define the precise structural or energetic basis for these observations. Future studies integrating structural analysis, enzyme kinetics, mutagenesis, and computational modeling will therefore be important for clarifying how proton concentration reshapes lesion recognition and strand selection in this enzyme family. Some strand-preference ratios calculated under strongly acidic or alkaline conditions should also be interpreted cautiously because overall repair efficiencies for certain enzyme–substrate combinations approached baseline levels (<5%). Under these low-activity conditions, small variations in product amount can produce large variability in the calculations of ssDNA/dsDNA fold-difference, consistent with the comparatively large SEM values observed at these pH points. Consequently, strand-preference trends observed within high-activity pH ranges are likely more reliable than those measured under low-activity conditions.

An additional observation worth noting was the comparatively high activity of ALKBH3 on ss-and dsDNA containing 3mC at pH 3.5 (Figure 5c, Tables 3 and S9), despite strongly reduced activity for most other enzyme–substrate combinations under similarly acidic conditions. Although low pH would generally be expected to impair Fe­(II)/2-oxoglutarate-dependent catalysis through protonation of 2-oxoglutarate (p*K*_a_ values = 2.2 and 4.6), this could affect 2-OG binding, oxygen activation, and enzyme catalysis irrespective of the substrate. However, repeated analyses confirmed that this behavior was reproducible across independent experiments. While we currently do not have a clear mechanistic explanation for this behavior, the observation appears reproducible rather than artifactual and may warrant further investigation in future mechanistic or enzyme-engineering studies.

## Conclusion

Overall, this study supports a revised view of AlkB-family catalysis in which pH acts as a condition-dependent chemical factor that can preserve, weaken, or reshape strand preference depending on the enzyme–substrate pair. These findings demonstrate that proton concentration influences catalytic efficiency, substrate utilization, and strand behavior under defined biochemical conditions, improving our understanding of AlkB-family enzyme function and aiding the design of biochemical assays and engineered applications.

## Supplementary Material



## Notes

Reported substrate scope of AlkB and human AlkB homologs (ALKBH2 and ALKBH3) (Table S1), calculated and observed monoisotopic molecular weight and m/z values of oligonucleotides used in the enzyme-pH activity reactions (Table S2), enzyme concentrations used for each substrate (Table S3), fold-difference analysis of AlkB, ALKBH2, and ALKBH3 activity on 1mA and 3mC-containing ssDNA and dsDNA across various pH (Tables S4-S9), SDS-PAGE analyses of AlkB-family proteins expression and purification (Figure S1), titration profile of the universal buffer system (Figure S2), LC–MS confirmation of 1mA demethylation by AlkB-family enzymes (Figure S3), HPLC identification of substrates and reaction products by retention time (Figure S4), HPLC and LC–MS characterization of synthesized oligonucleotides (Figure S5), and HPLC analyses of AlkB, ALKBH2 and ALKBH3 activities on dsDNA substrates across different pH conditions (Figure S6) (PDF)
