# Supplementary material for: pH Tunes the DNA Repair Efficiency and Strand Preference of the AlkB Family Enzymes
Source: Chem Res Toxicol. 2026 Jun 8;39(7):1418–30. doi: 10.1021/acs.chemrestox.6c00202 (PMC13390031; doi:10.1021/acs.chemrestox.6c00202)
Supplement: Supplementary file 1 [file tx6c00202_si_001.pdf]

SUPPORTING INFORMATION

pH Tunes the DNA Repair Efficiency and Strand Preference of the AlkB Family Enzymes

Samuel D. Howarth[1], Quentin J. Mylie[1], Evans Boateng-Boakye[1], Vincent Falkowski[1], Ambar Oliver Mella[1], Rafael Fermin[1] , Zhiyuan Peng[1], Xin Bush[1], Yi-Tzai Chen[1], Jian Ma[1], Bongsup Cho[1], Deyu Li[1,*]

[1] *Department of Biomedical and Pharmaceutical Sciences, College of Pharmacy, University of Rhode Island, Kingston, RI 02881, USA.*

[*]To whom correspondence should be addressed: deyuli@uri.edu

TABLE OF CONTENTS

Table S1. Reported substrate scope of AlkB and human AlkB homologs (ALKBH2 and ALKBH3).

Table S2. Calculated and observed monoisotopic molecular weight and *m*/*z* values of oligonucleotides used in the enzyme-pH activity reactions.

Table S3. Enzyme concentrations used for each substrate.

Table S4. Fold-difference analysis of AlkB activity on 1mA-containing ssDNA and dsDNA across different pH conditions.

Table S5. Fold-difference analysis of AlkB activity on 3mC-containing ssDNA and dsDNA across different pH conditions.

Table S6. Fold-difference analysis of ALKBH2 activity on 1mA-containing ssDNA and dsDNA across different pH conditions.

Table S7 Fold-difference analysis of ALKBH2 activity on 3mC-containing ssDNA and dsDNA across different pH conditions.

Table S8. Fold-difference analysis of ALKBH3 activity on 1mA-containing ssDNA and dsDNA across different pH conditions.

Table S9. Fold-difference analysis of ALKBH3 activity on 3mC-containing ssDNA and dsDNA across different pH conditions.

Figure S1. SDS–PAGE analysis of AlkB-family protein expression and purification.

Figure S2. Titration profile of the universal buffer system.

Figure S3. LC–MS confirmation of 1mA demethylation by AlkB-family enzymes.

Figure S4. HPLC identification of substrates and reaction products by retention time.

Figure S5. HPLC and LC–MS characterization of synthesized oligonucleotides.

Figure S6. HPLC analyses of AlkB, ALKBH2 and ALKBH3 activities on dsDNA substrates across different pH conditions.

Table S1. Reported substrate scope of AlkB and human AlkB homologs (ALKBH2 and ALKBH3). Known DNA and RNA substrates of *E. coli* AlkB, ALKBH2, and ALKBH3. AlkB shows broad substrate scope, whereas ALKBH2 and ALKBH3 are more selective, with ALKBH2 favoring dsDNA lesions and ALKBH3 preferring ssDNA and RNA substrates. This summary provides context for the substrates examined in this study.

Enzyme	Substrate
AlkB	**DNA**: 1mA, 3mC, 1mG, 3mT, 4mC, 2mG, 5mC, e^{1}A, εA, εC, 1,N^2-εG, e2G, EA, FF, HF, αHOPG, γHOPG, M1G, HEC, HPC
	**RNA**: m1A, m3C, m1G, m22G
ALKBH2	**DNA**: 1mA, 3mC, 1mG, 3mT, 5mC, 1eA, 3eT, εA, εC, 1,N^2-εG
ALKBH3	**DNA**: 1mA, 3mC, 3mT, 5mC, 1eA, 3eT, εC, εA
	**RNA**: m1A, m3C, m6A, m1,6A

Table S2. Calculated and observed monoisotopic molecular weight and *m*/*z* values of oligonucleotides used in the enzyme-pH activity reactions. The sequence of the 16mer oligos is 5'-GAAGACCTXGGCGTCC-3', where X indicates the position of the modification and the repaired products. The sequence of the 23mer oligos is 5'-CTGGGACGCCYAGGTCTTCACTG-3', where Y represents the position incorporating the complementary bases.

Modification	MW (calculated) of neutral species	m/z (calculated) -3 charge monoisotopic peak	m/z (observed) -3 charge monoisotopic peak	m/z (calculated) -2 charge monoisotopic peak	m/z (observed) -2 charge monoisotopic peak
16mer 1mA	4902.8769	1633.2845	1633.2864	2450.4306	2450.4291
16mer A	4888.8612	1628.6126	1628.6153	2443.4228	2443.4215
16mer 3mC	4878.8657	1625.2807	1625.2746	2438.4250	2438.4217
16mer C	4864.8500	1620.6088	1620.6110	2431.4172	2431.4144
23mer T	7028.1856	2341.7207	2341.7265	3513.0850	3513.0780
23mer G	7053.1921	2350.0562	2350.0527	3525.5882	3525.5837

Table S3. Enzyme concentrations used for each substrate. Final concentrations of AlkB, ALKBH2, and ALKBH3 used for in vitro oxidative reactions with 1mA, and 3mC in ssDNA and dsDNA.

Modification (ssDNA and dsDNA)	AlkB (μM)	ALKBH2 (μM)	ALKBH3 (μM)
1mA	0.25	0.31	0.31
3mC	0.25	0.31	0.31

Table S4. Fold-difference analysis of AlkB activity on 1mA-containing ssDNA and dsDNA across different pH conditions. Repair efficiencies for ssDNA and dsDNA substrates are shown as mean ± standard error of the mean (SEM) (n = 4 independent reactions), together with the calculated fold-difference (ssDNA/dsDNA) and propagated SEM at each pH value. Fold-differences greater than 1 indicate higher activity on ssDNA, whereas values less than 1 indicate higher activity on dsDNA. This shows that AlkB exhibited its expected ssDNA preference for 1mA under acidic conditions, with ssDNA/dsDNA ratios of 2.58 ± 0.09 at pH 4.0 and 1.61 ± 0.16 at pH 4.5, but this preference was lost at higher pH, where activity shifted toward dsDNA.

pH	Repair Efficiency (ssDNA)	Repair Efficiency (dsDNA)	Fold-difference (ssDNA/dsDNA)
3.5	1.16 ± 0.04	1.67 ± 0.76	0.70 ± 0.32
4.0	9.15 ± 0.12	3.55 ± 0.12	2.58 ± 0.09
4.5	23.89 ± 1.74	14.83 ± 0.99	1.61 ± 0.16
5.0	17.51 ± 0.35	21.50 ± 0.20	0.81 ± 0.02
5.5	18.35 ± 0.15	26.20 ± 0.74	0.70 ± 0.02
6.0	25.97 ± 0.53	42.92 ± 0.84	0.61 ± 0.02
6.5	51.74 ± 0.79	59.43 ± 0.94	0.87 ± 0.02
7.0	56.15 ± 0.61	77.00 ± 0.92	0.73 ± 0.01
7.5	62.76 ± 2.69	73.03 ± 0.25	0.86 ± 0.04
8.0	31.68 ± 2.49	60.32 ± 0.55	0.53 ± 0.04
8.5	28.41 ± 2.05	45.18 ± 0.56	0.63 ± 0.05
9.0	12.74 ± 3.03	16.96 ± 0.21	0.75 ± 0.18

Table S5. Fold-difference analysis of AlkB activity on 3mC-containing ssDNA and dsDNA across different pH conditions. Repair efficiencies for ssDNA and dsDNA substrates are shown as mean ± standard error of the mean (SEM) (n = 4 independent reactions), together with the calculated fold-difference (ssDNA/dsDNA) and propagated SEM at each pH value. Fold-differences greater than 1 indicate higher activity on ssDNA, whereas values less than 1 indicate higher activity on dsDNA. AlkB maintained ssDNA preference for 3mC across the entire pH range tested, with fold-differences consistently greater than 1. These findings indicate that proton concentration does not uniformly affect AlkB strand preference but instead modulates strand bias in a lesion-specific manner.

pH	Repair Efficiency (ssDNA)	Repair Efficiency (dsDNA)	Fold-difference (ssDNA/dsDNA)
3.5	5.19 ± 0.14	1.99 ± 0.43	2.61 ± 0.58
4.0	10.64 ± 0.07	4.47 ± 0.53	2.38 ± 0.28
4.5	10.12 ± 0.22	5.94 ± 0.22	1.70 ± 0.10
5.0	10.04 ± 0.36	8.81 ± 0.15	1.14 ± 0.05
5.5	14.43 ± 0.09	12.39 ± 0.78	1.16 ± 0.08
6.0	20.72 ± 0.88	15.37 ± 0.93	1.35 ± 0.10
6.5	41.36 ± 0.55	22.79 ± 0.74	1.81 ± 0.08
7.0	68.15 ± 0.32	35.15 ± 0.39	1.94 ± 0.03
7.5	69.32 ± 1.12	33.88 ± 0.56	2.05 ± 0.05
8.0	70.49 ± 0.84	33.15 ± 0.53	2.13 ± 0.04
8.5	42.31 ± 1.10	21.48 ± 0.11	1.97 ± 0.05
9.0	30.84 ± 0.33	20.88 ± 0.64	1.48 ± 0.05

Table S6. Fold-difference analysis of ALKBH2 activity on 1mA-containing ssDNA and dsDNA across different pH conditions. Repair efficiencies for ssDNA and dsDNA substrates are shown as mean ± standard error of the mean (SEM) (n = 4 independent reactions), together with the calculated fold-difference (ssDNA/dsDNA) and propagated SEM at each pH value. Fold-differences greater than 1 indicate higher activity on ssDNA, whereas values less than 1 indicate higher activity on dsDNA. ALKBH2 consistently favored dsDNA over ssDNA for 1mA across the entire pH range tested, with ssDNA/dsDNA ratios remaining below 1 at all pH values.

pH	Repair Efficiency (ssDNA)	Repair Efficiency (dsDNA)	Fold-difference (ssDNA/dsDNA)
3.5	1.06 ± 0.05	1.78 ± 0.14	0.60 ± 0.04
4.0	1.37 ± 0.26	2.39 ± 0.05	0.57 ± 0.11
4.5	1.63 ± 0.05	9.36 ± 0.14	0.17 ± 0.01
5.0	8.45 ± 0.06	12.78 ± 0.65	0.66 ± 0.03
5.5	14.02 ± 0.22	17.01 ± 0.39	0.82 ± 0.02
6.0	22.31 ± 1.74	31.37 ± 0.57	0.71 ± 0.06
6.5	35.12 ± 1.97	57.67 ± 1.09	0.61 ± 0.04
7.0	38.21 ± 0.35	71.50 ± 0.15	0.53 ± 0.01
7.5	15.98 ± 0.41	49.79 ± 1.60	0.32 ± 0.01
8.0	8.12 ± 0.17	26.95 ± 0.68	0.30 ± 0.01
8.5	5.20 ± 0.07	20.35 ± 0.07	0.26 ± 0.00
9.0	4.98 ± 0.18	19.29 ± 0.42	0.26 ± 0.01

Table S7 Fold-difference analysis of ALKBH2 activity on 3mC-containing ssDNA and dsDNA across different pH conditions. Repair efficiencies for ssDNA and dsDNA substrates are shown as mean ± standard error of the mean (SEM) (n = 4 independent reactions), together with the calculated fold-difference (ssDNA/dsDNA) and propagated SEM at each pH value. Fold-differences greater than 1 indicate higher activity on ssDNA, whereas values less than 1 indicate higher activity on dsDNA. Unlike 1mA, ALKBH2 exhibited a clear pH-dependent shift in strand preference for 3mC, favoring ssDNA under acidic to mildly acidic conditions before transitioning toward dsDNA preference at neutral to alkaline pH.

pH	Repair Efficiency (ssDNA)	Repair Efficiency (dsDNA)	Fold-difference (ssDNA/dsDNA)
3.5	1.36 ± 0.05	1.72 ± 0.20	0.79 ± 0.09
4.0	6.92 ± 0.04	3.2 ± 0.17	2.16 ± 0.11
4.5	32.67 ± 0.29	8.56 ± 0.65	3.82 ± 0.30
5.0	49.08 ± 0.67	11.42 ± 0.16	4.30 ± 0.07
5.5	62.75 ± 2.15	21.13 ± 0.84	2.97 ± 0.12
6.0	75.13 ± 0.84	42.99 ± 3.0	1.75 ± 0.13
6.5	75.03 ± 0.24	49.17 ± 0.42	1.53 ± 0.02
7.0	46.77 ± 0.15	48.21 ± 0.22	0.97 ± 0.00
7.5	23.61 ± 3.24	36.38 ± 0.46	0.65 ± 0.09
8.0	3.97 ± 0.036	16.70 ± 0.42	0.24 ± 0.01
8.5	3.08 ± 0.039	8.69 ± 0.44	0.35 ± 0.02
9.0	2.80 ± 0.00	10.65 ± 0.11	0.26 ± 0.00

Table S8. Fold-difference analysis of ALKBH3 activity on 1mA-containing ssDNA and dsDNA across different pH conditions. Repair efficiencies for ssDNA and dsDNA substrates are shown as mean ± standard error of the mean (SEM) (n = 4 independent reactions), together with the calculated fold-difference (ssDNA/dsDNA) and propagated SEM at each pH value. Fold-differences greater than 1 indicate higher activity on ssDNA, whereas values less than 1 indicate higher activity on dsDNA. ALKBH3 exhibited a non-uniform pH-dependent strand preference for 1mA, alternating between ssDNA- and dsDNA-favored activity across the tested pH range.

pH	Repair Efficiency (ssDNA)	Repair Efficiency (dsDNA)	Fold-difference (ssDNA/dsDNA)
3.5	2.36 ± 0.41	1.29 ± 0.04	1.83 ± 0.32
4.0	6.60 ± 0.36	1.68 ± 0.02	3.93 ± 0.21
4.5	32.76 ± 1.02	50.27 ± 0.76	0.65 ± 0.02
5.0	35.59 ± 0.56	89.82 ± 0.06	0.40 ± 0.01
5.5	34.52 ± 0.09	89.98 ± 0.16	0.38 ± 0.00
6.0	47.79 ± 0.29	84.22 ± 0.12	0.57 ± 0.00
6.5	64.92 ± 0.44	60.83 ± 0.34	1.07 ± 0.01
7.0	38.85 ± 0.76	20.30 ± 0.14	1.91 ± 0.04
7.5	16.02 ± 0.26	4.78 ± 0.04	3.35 ± 0.06
8.0	7.57 ± 0.24	5.23 ± 0.04	1.45 ± 0.05
8.5	5.37 ± 0.05	6.08 ± 0.19	0.88 ± 0.03
9.0	4.66 ± 0.01	6.40 ± 0.19	0.73 ± 0.02

Table S9. Fold-difference analysis of ALKBH3 activity on 3mC-containing ssDNA and dsDNA across different pH conditions. Repair efficiencies for ssDNA and dsDNA substrates are shown as mean ± standard error of the mean (SEM) (n = 4 independent reactions), together with the calculated fold-difference (ssDNA/dsDNA) and propagated SEM at each pH value. Fold-differences greater than 1 indicate higher activity on ssDNA, whereas values less than 1 indicate higher activity on dsDNA. ALKBH3 consistently favored ssDNA over dsDNA for 3mC across the entire pH range tested.

pH	Repair Efficiency (ssDNA)	Repair Efficiency (dsDNA)	Fold-difference (ssDNA/dsDNA)
3.5	48.87 ± 0.30	6.08 ± 0.43	8.05 ± 0.58
4.0	78.92 ± 1.64	7.85 ± 0.50	10.05 ± 0.67
4.5	82.25 ± 0.86	31.42 ± 0.13	2.62 ± 0.03
5.0	85.36 ± 1.11	32.21 ± 0.43	2.65 ± 0.05
5.5	81.97 ± 0.86	27.17 ± 0.16	3.02 ± 0.03
6.0	93.08 ± 0.99	29.06 ± 0.65	3.20 ± 0.08
6.5	85.49 ± 1.94	17.86 ± 0.31	4.79 ± 0.12
7.0	81.29 ± 0.92	8.59 ± 0.20	9.47 ± 0.27
7.5	46.17 ± 0.06	3.37 ± 0.16	13.83 ± 0.69
8.0	17.45 ± 1.07	3.40 ± 0.00	5.13 ± 0.31
8.5	9.73 ± 0.33	3.90 ± 0.00	2.49 ± 0.08
9.0	8.62 ± 0.21	4.40 ± 0.03	1.96 ± 0.05

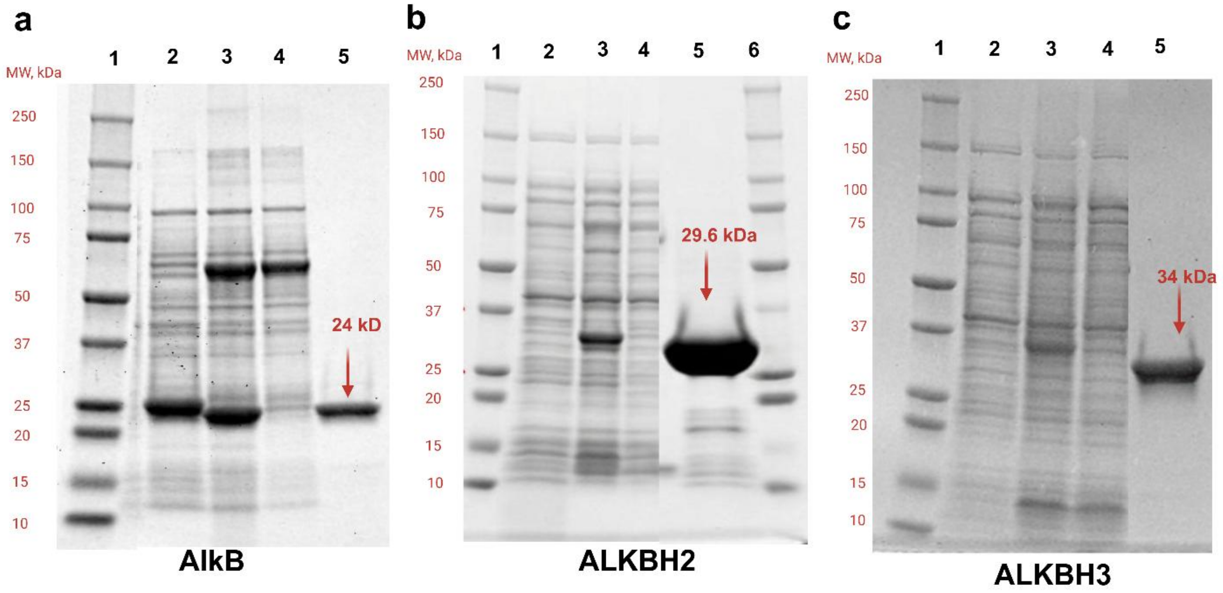

Figure S1. SDS–PAGE analysis of AlkB-family protein expression and purification. Coomassie-stained SDS–PAGE gels showing expression and purification of AlkB, ALKBH2, and ALKBH3. Lanes 1 and 6: molecular weight marker. Lanes 2–4: intermediate stages of expression and purification. Lane 5: purified proteins, with bands corresponding to AlkB (~24 kDa), ALKBH2 (~29.6 kDa), and ALKBH3 (~34 kDa).

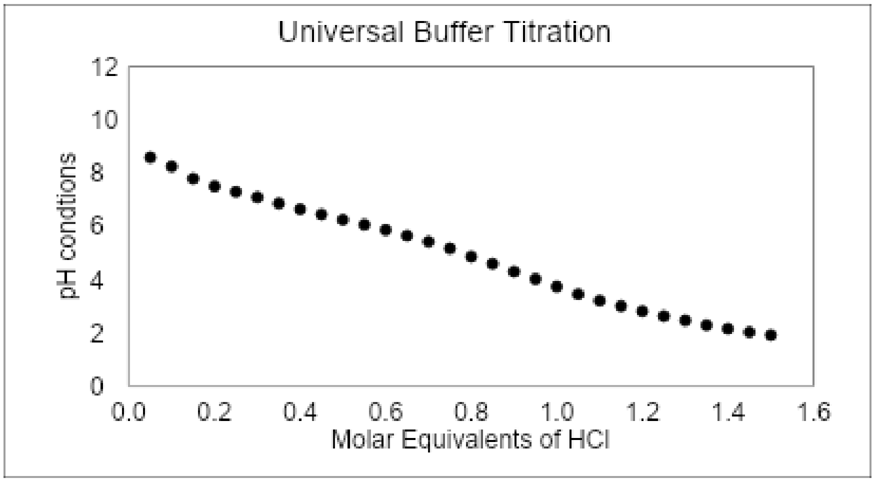

Figure S2. Titration profile of the universal buffer system. pH response of the MES/HEPES/sodium acetate buffer mixture upon titration with HCl. Incremental addition of acid (expressed as molar equivalents of HCl) results in a gradual decrease in pH, demonstrating the buffering capacity of the system across the pH range used in this study.

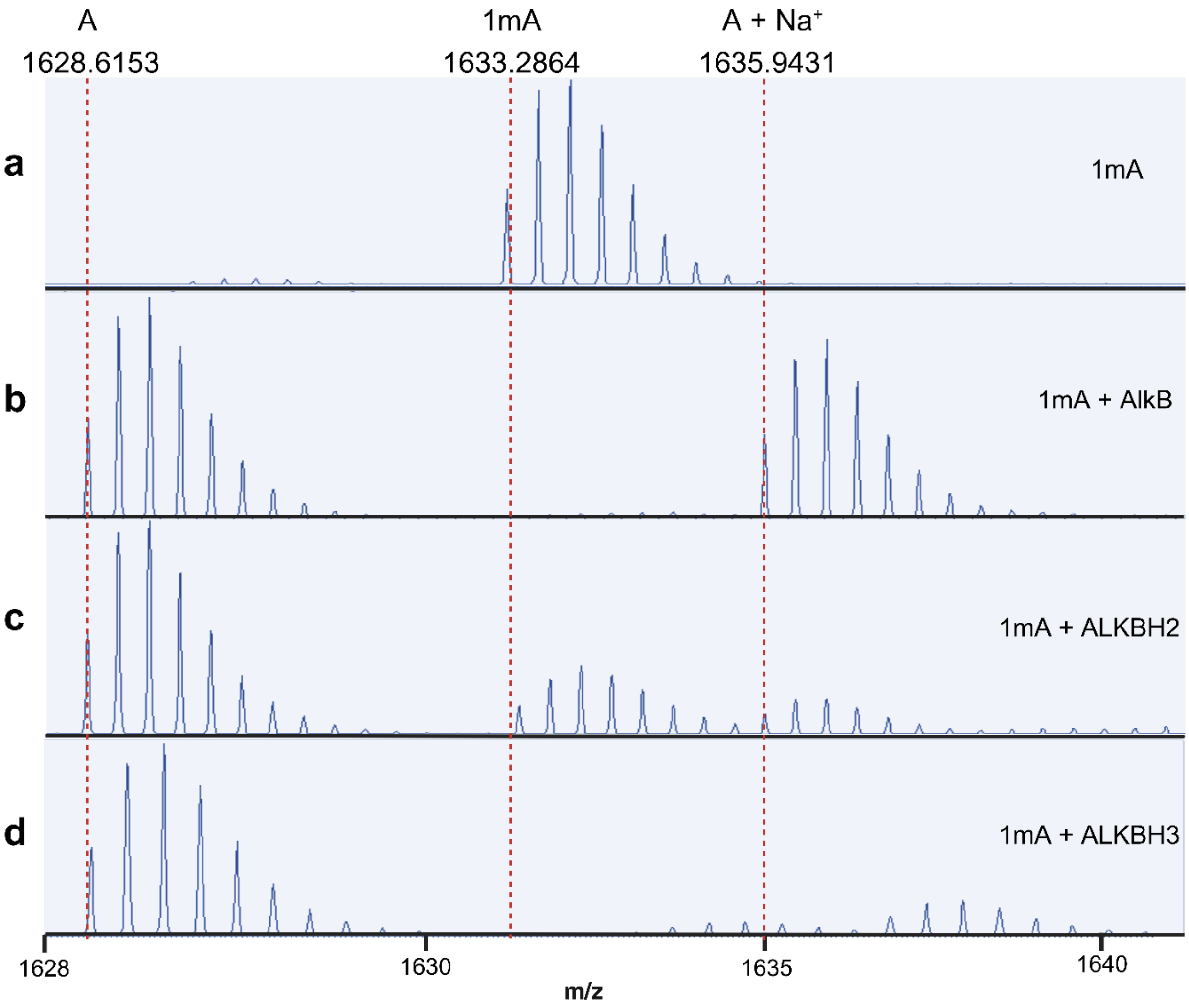

Figure S3. LC–MS confirmation of 1mA demethylation by enzymes. (a) Mass spectrum of the 1mA-containing oligonucleotide showing the expected *m*/*z* for the substrate. (b–d) Mass spectra following treatment with AlkB, ALKBH2, and ALKBH3, respectively. The appearance of the demethylated product (A) and corresponding sodium adduct, with a concomitant decrease in the 1mA signal, confirms enzymatic demethylation. Dashed lines indicate expected m/z values for substrate and product species.

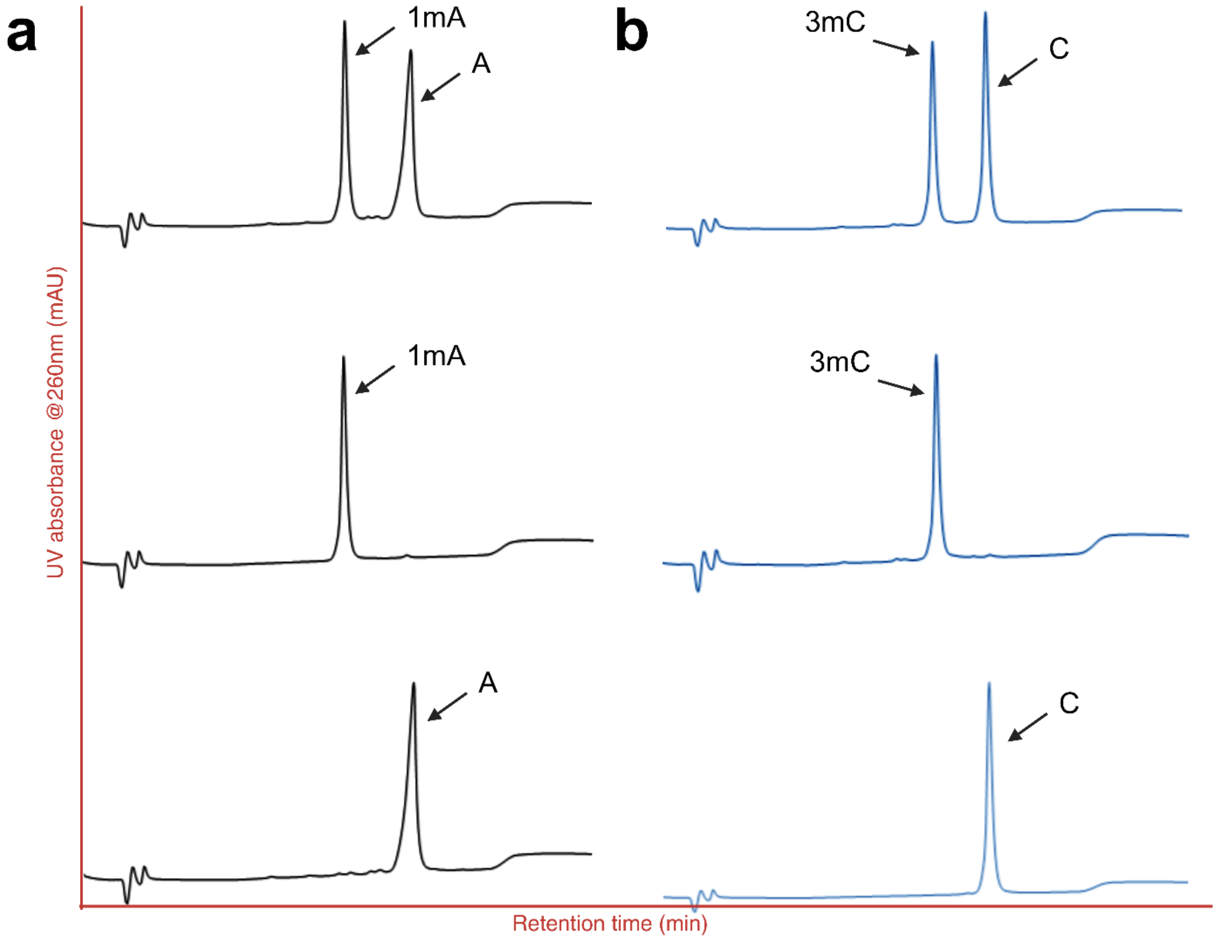

Figure S4. HPLC identification of substrates and reaction products by retention time. Representative HPLC traces of intact oligonucleotides containing 1mA (a), 3mC (b), and their corresponding product standards. The single, well-resolved peaks for the starting materials confirm substrate purity, while distinct retention times for substrate and product species enabled assignment of reaction products in enzymatic assays.

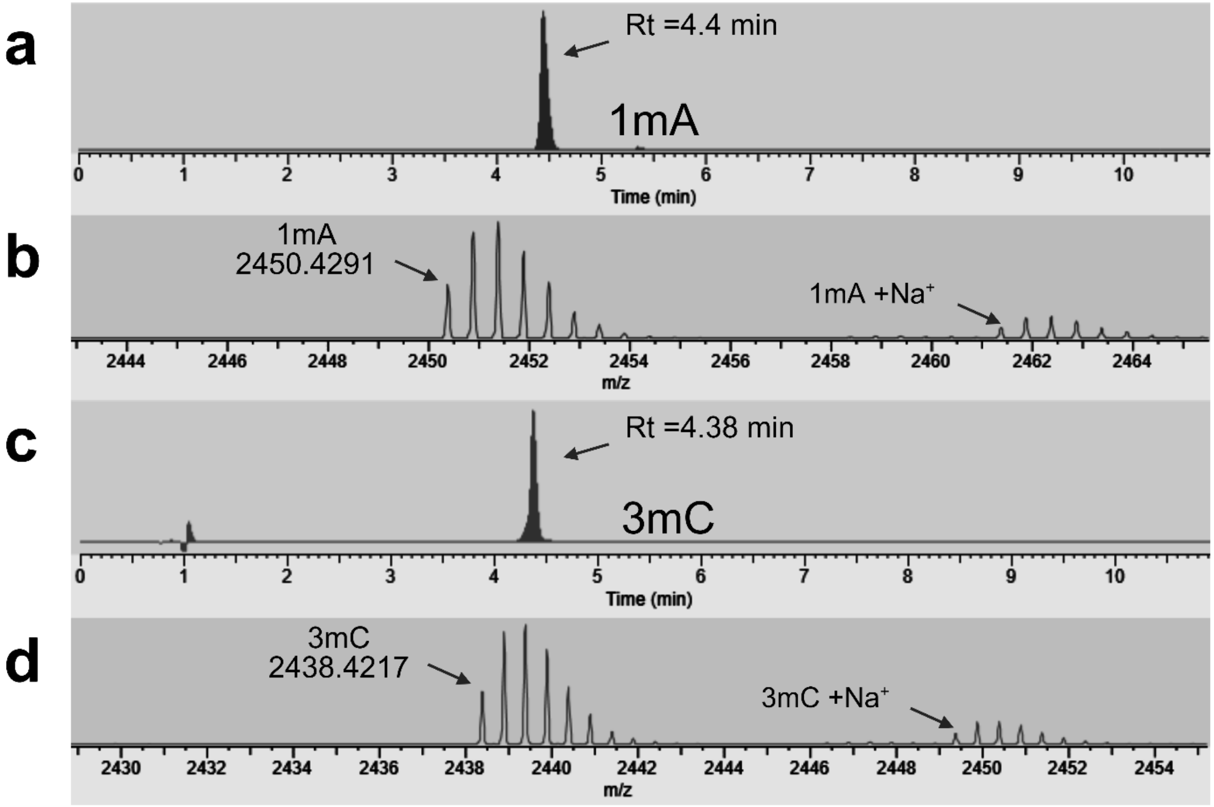

Figure S5. HPLC and LC–MS characterization of synthesized oligonucleotides. (a, c) HPLC UV traces of purified oligonucleotides containing 1mA, and 3mC respectively, showing single, well-resolved peaks indicative of high purity. (b, d) Corresponding mass spectra of the HPLC peaks at the −2 charge state, confirming the expected *m*/*z* values for each oligonucleotide and their sodium adducts. Together, these data verify the identity and purity of the substrates used in this study.

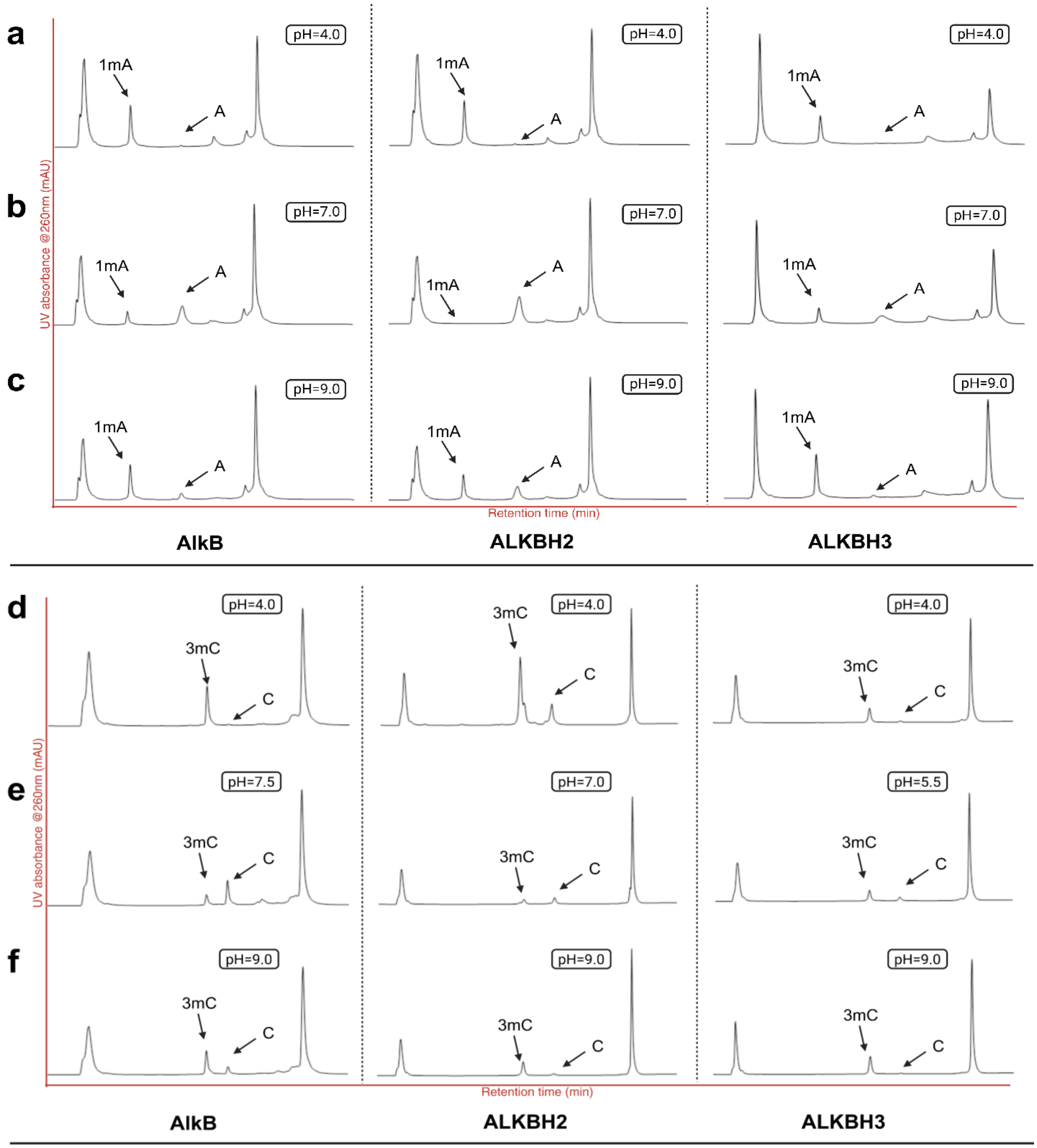

Figure S6. HPLC analyses of AlkB, ALKBH2 and ALKBH3 activities on dsDNA substrates across different pH conditions. Representative HPLC traces showing oxidative demethylation of dsDNA substrates by AlkB, ALKBH2, and ALKBH3 at different pH conditions. (a–c) 1mA-containing dsDNA and formation of A. (d–f) 3mC-containing dsDNA and formation of C. Peaks corresponding to substrate and product are labeled, illustrating enzyme- and pH-dependent activity.
